# Isolated Infiltrative Optic Neuropathy in an Acute Lymphoblastic Leukemia Relapse

**DOI:** 10.7759/cureus.25625

**Published:** 2022-06-03

**Authors:** Huey Chuin Kuan, Mushawiahti Mustapha, Shelina Oli Mohamed, Roslin Azni Abdul Aziz, C Khai Loh, Fazarina Mohammed, Ainal Adlin Naffi, Othmaliza Othman, Rona A Nasaruddin, Hamidah Alias

**Affiliations:** 1 Ophthalmology, Hospital Universiti Kebangsaan Malaysia, Kuala Lumpur, MYS; 2 Ophthalmology, Hospital Shah Alam, Shah Alam, MYS; 3 Pediatric Oncology, Hospital Universiti Kebangsaan Malaysia, Kuala Lumpur, MYS; 4 Pathology, Hospital Universiti Kebangsaan Malaysia, Kuala Lumpur, MYS; 5 Ophthalmology, Universiti Kebangsaan Malaysia Medical Centre, Kuala Lumpur, MYS

**Keywords:** leukemia, retina artery occlusion, retina vein occlusion, optic disc, optic neuropathy

## Abstract

Optic nerve infiltration as the first sign of isolated central nervous system relapse of acute lymphoblastic leukemia (ALL) is rare. A seven-year-old girl with standard-risk B-cell ALL who was in remission presented with sudden onset of left eye pain and loss of vision. Examination revealed no perception to light in the left eye with positive relative afferent pupillary defect. The optic disc was hyperemic and swollen with total obscuration of the disc margin associated with central retinal artery and vein occlusion. Magnetic resonance imaging of the brain and optic nerve showed left intraorbital optic nerve thickening associated with perineural enhancement and intraconal fat involvement.

Lumbar puncture revealed leukemic infiltration with blast cells after a week of eye symptoms, while bone marrow aspiration was negative for malignant cells. A diagnosis of left leukemic optic nerve infiltration with central retinal artery and vein occlusion was made. A high index of suspicion with repeat cerebrospinal fluid sampling is crucial to confirm the diagnosis as vitreous biopsy may fail to reveal infiltrative cells.

## Introduction

Optic nerve involvement is reported to occur in one-sixth to 34% of leukemic cases [1,2]. However, isolated central nervous system (CNS) relapse in acute lymphoblastic leukemia (ALL) with optic nerve infiltration as the initial presentation is rare. The majority of leukemic optic nerve involvement occurs in patients with acute bone marrow disease [1,3]. Optic nerve infiltration may be the first presenting sign of acute leukemic relapse before the hematological involvement [3]. We report a rare case of ALL relapse presenting with optic nerve infiltration associated with central retina vein and artery occlusion without evidence of hematological relapse.

This article was previously presented as a meeting poster at the 33rd Asia-Pacific Association of Cataract & Refractive Surgeons (APACRS)-Singapore National Eye Centre (SNEC) 30th Anniversary Virtual Meeting on July 30-31, 2021.

## Case presentation

A seven-year-old girl with standard-risk B-cell ALL diagnosed two years ago who had completed her systemic and prophylactic intrathecal chemotherapy and was in complete remission for two months presented with sudden onset of left eye pain and loss of vision. It occurred upon awakening from sleep and was associated with throbbing headaches. She did not have any complaint over the right eye. She had no history of hyperleucocytosis, extramedullary disease, or CNS involvement during her first diagnosis of ALL. Systematically, there was no preceding fever or signs of systemic infection.

**Figure 1:**
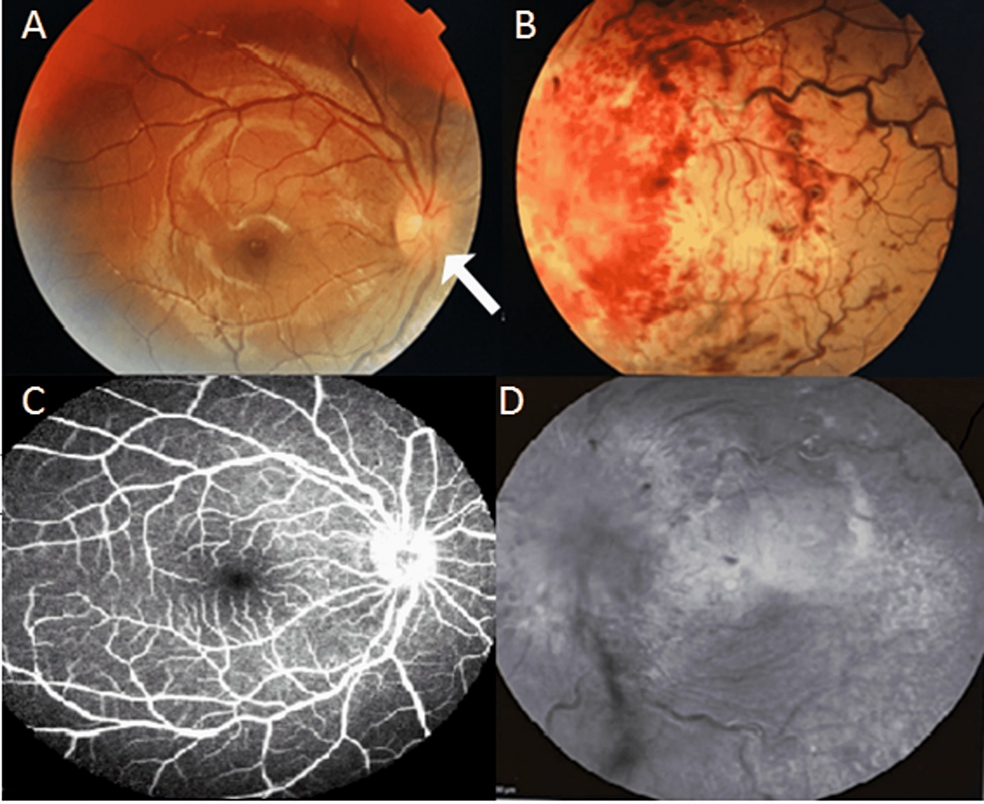
(A) Fundus image showing the right optic disc margin with a blurred nasal margin (arrow) and (B) generalized retinal hemorrhages with total obscuration of the left optic disc margin, with dilated and tortuous retinal veins. (C) Fundus fluorescein of the right eye showing a hot disc. (D) Fundus fluorescein angiography of the left eye showing total absence filling of the choroid, retina artery, and vein for up to seven minutes.

Her left visual acuity was non-perception to light with positive relative afferent pupillary defect. Right visual acuity was 3/3 on the Sheridan Gardiner test. Anterior segment was unremarkable in both eyes. Left eye fundus examination revealed a markedly swollen, hyperemic optic disc bulging into the vitreous with total obscuration of the disc margin. Retinal veins appeared very dilated and tortuous with presence of pale, edematous macula and retina. There were flame-shaped intraretinal hemorrhages in all four quadrants with vitreous hemorrhage inferiorly. There were no cotton wool spots (Figure 1, Panel B). Her right eye fundus showed sectoral blurred optic disc margin nasally (Figure 1, Panel A). Otherwise, the retina was normal. A clinical diagnosis of left leukemic optic nerve infiltration with central retinal artery and vein occlusion was made.

**Figure 2:**
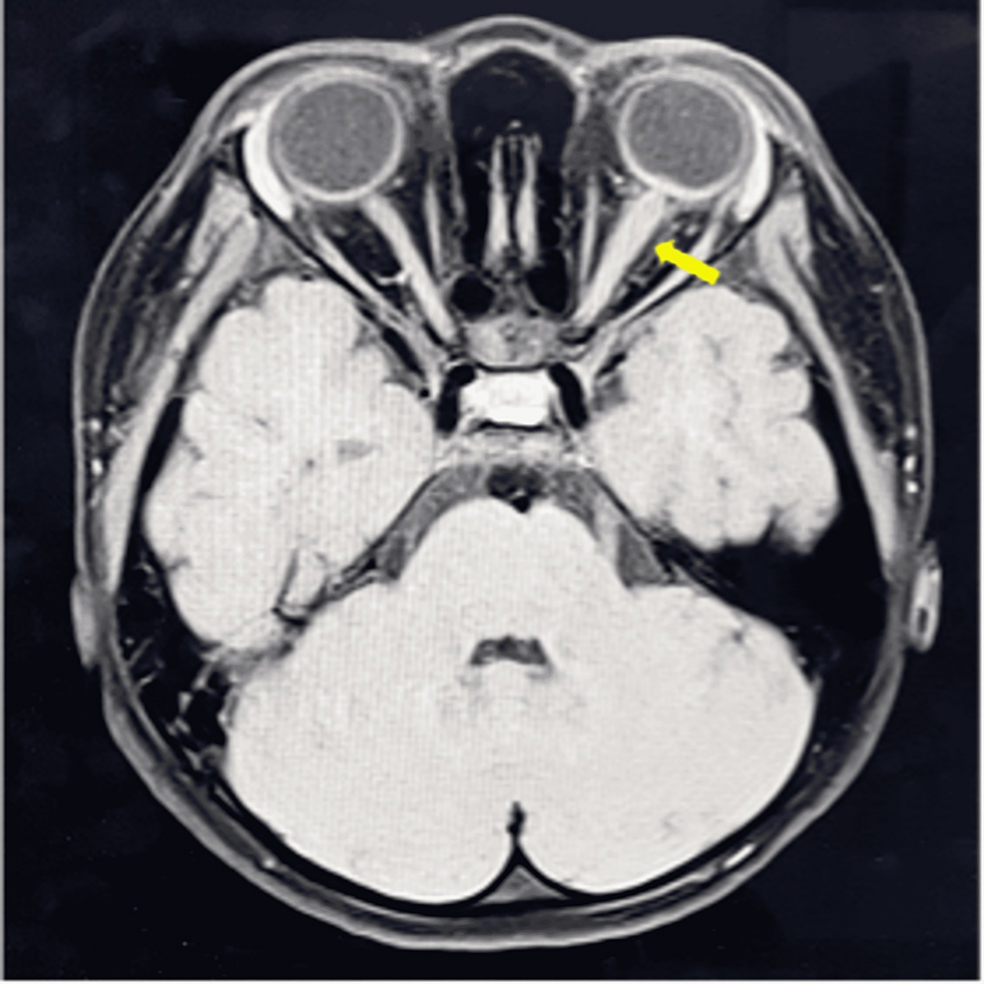
MRI T1-weighted brain and optic nerve revealing left intraorbital optic nerve thickening (yellow arrow) associated with perineural enhancement and intraconal fat involvement on DWI/ADC (DWI/ADC image is not included in this figure). DWI: diffusion-weighted imaging; ADC: apparent diffusion coefficient

An urgent magnetic resonance imaging (MRI) of the brain and optic nerve showed left intraorbital optic nerve thickening associated with perineural enhancement and intraconal fat involvement (Figure 2). The right optic nerve was normal with an absence of enhancement or fat stranding. Otherwise, no abnormal leptomeningeal enhancement or features suggestive of CNS leukemia or increased intracranial pressure were noted.

Fundus fluorescein angiography revealed a complete absence of choroidal, retinal artery, and venous filling of the left eye for up to seven minutes while the right eye showed leakage from the optic disc (Figure 1, Panels C and D), signifying likely bilateral eye involvement. Optical coherence tomography of both maculae showed hyperreflective vitreous dots with the presence of thickened retina in the left eye.

**Figure 3:**
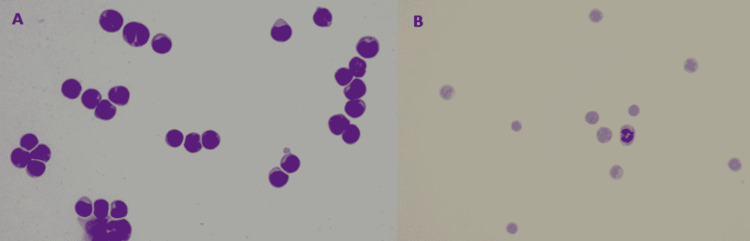
(A) Cytospinned sediment of cerebrospinal fluid shows numerous blast cells with high nuclear-to-cytoplasmic ratio and conspicuous nucleoli (MGG, 40×) and (B) mitosis (Pap stain, 40×). MGG: May Grunwald-Giemsa; Pap: Papanicolaou

Bone marrow aspiration and trephine biopsy (BMAT) and initial lumbar puncture (LP) were negative for malignancy. Second LP was done a week later together with anterior chamber tapping and vitreous biopsy. Cerebrospinal fluid cytology showed presence of blast cells, suggestive of leukemic infiltration of the CNS (Figures 3A, 3B). Anterior chamber tapping and vitreous biopsy were negative for malignancy. Repeat BMAT showed reactive lymphocytosis with no excess blast noted.

In view of relapse of leukemia with isolated CNS involvement, she was started on reinduction chemotherapy UK ALL R3 including intrathecal methotrexate. Neither local ocular chemotherapy nor radiotherapy was initiated. To date, her left eye remains without perception to light with right eye vision maintained without deterioration to date.

## Discussion

ALL is an aggressive malignant proliferation of lymphoblasts with peak prevalence between the ages of one and four [4]. It can invade blood, bone marrow, and extramedullary sites including the CNS. Treatment regimens are based on risk stratification and prognostic factors which include age, gender, ethnicity, blood count at diagnosis, cell lineage, and CNS involvement [4].

Optic nerve is known to be a sanctuary for leukemic cells which is relatively unaffected by systemic chemotherapy and even intrathecal chemotherapy [5]. Cases of both unilateral and bilateral optic nerve involvement have been reported [6,7].

In our case, despite intrathecal chemotherapy, which was given for CNS prophylaxis at the first diagnosis of ALL, and achieving complete remission after completion of chemotherapy, she developed isolated CNS relapse.

The majority of patients with CNS relapse present with symptoms such as altered mental status, headache, and cranial nerve palsy while only a minority (6.5%) report blurred vision as the initial complaint [8]. This was seen in our case where the patient presented with sudden loss of vision with headaches without other symptoms such as diplopia or altered mental state. Despite having unilateral eye complaint, fundoscopic examination revealed involvement of both optic nerves with early optic disc swelling in the contralateral eye.

Rosenthal classified optic nerve infiltration in leukemia into prelaminar and retrolaminar [9]. In prelaminar invasion, infiltrates occur superficially to the lamina cribosa and can be associated with moderate edema and hemorrhage of the optic nerve head. Visual acuity might be minimally affected unless there is co-existing macular edema and hemorrhage. Retrolaminar optic nerve invasion usually presents with significant disc elevation and edema with marked visual impairment. Our case had prelaminar and retrolaminar optic nerve invasion with total obscuration of the disc margin and significant visual loss and bulging of the optic disc into the vitreous, as evidenced by an MRI of the orbit. Nevertheless, the central retinal artery occlusion was believed to be the main contributing factor for the acute loss of vision rather than the optic nerve ischemia from the infiltrative optic neuropathy [5].

There are limited cases reported on central retinal vascular occlusion in ALL in contrast to other lymphoproliferative disorders such as lymphoma [10-12]. A case of pediatric age group leukemic optic neuropathy with sequential bilateral central retinal artery occlusion has been reported by Lin et al. [6] in which bilateral central retinal artery occlusion developed few hours apart. It has been postulated that vascular disturbances that occur in leukemic optic neuropathy are due to leukemic infiltration into or surrounding the blood vessels or secondary to neoplastic emboli [13].

A review by Myers et al. found that malignant cells are identified in the cerebrospinal fluid in a majority of leukemic infiltration cases [14]. In our case, the initial cerebrospinal fluid analysis was negative for blast cells and was only positive in a repeat examination performed a week later. A similar result occurred on immunophenotyping with flow cytometry where evidence of B-lymphoblastic infiltration in cerebrospinal fluid was only revealed in the repeat sample. Thus, a repeat investigation with fresh samples for detecting malignant cells might be indicated if there is a high index of suspicion for infiltrative optic neuropathy when the initial result does not support the diagnosis.

Vitreous biopsy has value in diagnosing a leukemic relapse when there is presence of dense vitreous cellular infiltration [15]. Shenoy et al. reported a similar case of isolated optic nerve relapse with cerebrospinal fluid analysis negative for blast cells which was confirmed by vitreous biopsy [16]. However, internal limiting membrane of the retina appears to be preserved from leukemic cell penetration, which, in turn, limits cell invasion into the vitreous [17]. This may explain our negative findings of blast cells in the vitreous sample, similar to a case reported by Bansal et al. [18]. Because of high risk and low yield, optic nerve biopsy is not recommended [19]. To date, there is no literature on the risk of tumor seeding in vitreous biopsy via trans par plana vitrectomy. On the other hand, the occurrence of tumor cells seeding in needle track following fine needle aspiration of biopsy of intraocular tumor varied from none to 54% [20,21]. Hence, benefits should outweigh the risk when considering vitreous biopsy.

## Conclusions

Optic nerve infiltration may be the only initial presentation indicating relapse of ALL following attainment of disease remission. A high index of suspicion and prompt diagnosis may not only avoid irreversible loss of vision but more importantly can be life-saving for the patient. Repeated cerebrospinal fluid analysis may be necessary in the event of initial negative results for malignancy.
